# Differential response of physiology and metabolic response to drought stress in different sweetpotato cultivars

**DOI:** 10.1371/journal.pone.0264847

**Published:** 2022-03-10

**Authors:** Zhilin Zhou, Jun Tang, Qinghe Cao, Zongyun Li, Daifu Ma

**Affiliations:** 1 School of Life Sciences, Jiangsu Normal University, Xuzhou, Jiangsu, China; 2 Xuzhou Institute of Agricultural Sciences in Xuhuai District, Xuzhou, Jiangsu, China; Northeast Forestry University, CHINA

## Abstract

Sweetpotato (*Ipomoea batatas* [L.] Lam) is a widely cultivated food crop with generally good adaptability. However, drought stress can cause a significant decline in yield. To reveal the response mechanism of sweetpotato to drought stress, an integrated physiological, proteomic and metabolomic investigation was conducted in leaves of two sweetpotato varieties with differing responses to drought stress, drought-resistant Wanzishu56 (WZ56) and a more sensitive variety, Ningzishu2(NZ2). Physiological analysis showed that the variety with better drought tolerance had superior performance in water retention capacity and photosynthetic efficiency under drought stress. A total of 1140 proteins were identified within the two varieties. Among them, 192 differentially expressed proteins were detected under drought conditions, including 97 that were up-regulated. Functional analysis showed that these up-regulated proteins were primarily involved in photosynthesis, reactive oxygen species metabolism, organonitrogen compound metabolism, and precursor metabolite catabolism and energy generation. All differentially expressed proteins in WZ56 that were involved in photosynthetic and glutathione metabolic processes were up-regulated. Enzyme activity assays were carried out to validate the proteomics data. Moreover, 75 metabolites were found to have a higher expression level in WZ56 than NZ2 under drought stress. The higher concentration of carbohydrates, amino acids, flavonoids and organic acids found in drought-stressed leaves of WZ56 suggested that these metabolites may improve the drought resistance of sweetpotato. This study uncovered specific-proteins and metabolites associated with drought resistance, providing new insights into the molecular mechanisms of drought tolerance in sweetpotato.

## Introduction

Drought stress is one of the major abiotic stress that limits plant growth and productivity worldwide. The effects of drought stress on plants are primarily characterized by water loss, reduced leaf water potential, turgor loss, stomata closure, and decreased cell elongation and growth [1]. Unpredictable drought conditions cause severe crop yield losses, threatening global food security [2]. In order to meet the needs of an increasing population, it is essential to effectively develop strategies for dryland farming in arid regions. In addition to usage of water conservancy facilities, development and implementation of drought-resistant crops is the most effective and practical method of fully utilizing water-scarce soil [3]. Thus, a comprehensive analysis of plant drought resistance mechanism would aid in this goal by contributing to rapid cultivation of drought-resistant crops.

With the development of advanced sequencing technology and bioinformatics tools, transcriptomic analyses have been used to parse drought stress responses in many crops, such as, sugarcane [4], potato [5], maize [6], and rice [7]. Results of such studies have indicated that differentially expressed genes were functionally associated with the drought-stress response [5] and mainly involved in energy metabolism, transport, antioxidants, photosynthesis, and stress signaling pathways [8]. In drought-tolerant genotype, a greater number of differentially expressed genes were associated with cell growth, hormone biosynthesis, cellular transport, amino acid metabolism, transcription factors, and carbohydrate metabolism [7].

However, many studies have indicated that post-transcriptional regulation may limit the correlation between expression levels of transcripts and their corresponding proteins [9–11]. Metabolomics and proteomics could therefore complement transcriptome data to provide more direct insight into metabolic processes.

Proteomics approaches to study drought resistance have been reported in cotton [12], common bean [13], citrus [14], wheat [15], soybean [16], and tobacco [17]. Proteins up-regulated in the stress-tolerant genotype under drought stress were found to be related to defense and oxidative stress responses [18]. Melanoma-associated antigen p97, type1 chlorophyll a/b b-binding protein b, glutathione S-transferase 1, and the ribulose bisphosphate carboxylase large chain were specifically expressed in drought-tolerant barley; these proteins may play an important role in barley under drought stress [19]. Metabolomics approaches have also been applied to study stress responses in Arabidopsis [20], rice [21] and wheat [22]. In these species, comparative metabolomics and proteomics have effectively facilitated the study of drought resistance mechanism.

As a staple food crop, sweetpotato (*Ipomoea batatas* [L.] Lam) is widely cultivated in Asia and Africa, typically under drought conditions. However, drought stress is one of the major challenges for sweetpotato production. In order to improve the drought resistance of sweetpotato, transgenic plants have been generated that overexpress endogenous or exogenous genes [23–26]. In addition to genetic engineering, some sweetpotato cultivars have shown naturally superior drought tolerance based on storage root yield; in a study of 84 sweetpotato cultivars evaluated by relative yield and drought stress index difference, four lines (19455537, W119, Tanzania, Chingova)were identified as drought-resistant [27]. Transcriptomics and proteomics analyses of storage root formation and storage stress responses have been reported in sweetpotato [28–31]. However, there is currently little information about the metabolic response to drought in sweetpotato with respect to proteomics and metabolomics. Therefore, we conducted a comparative integrated analysis of physiology, proteomics and metabolomics in the leaves of two sweetpotato varieties with different levels of drought resistance, WZ56 and NZ2, in response to drought. This research provided important practical guidance for the breeding and popularization of drought-tolerant sweetpotato and offers a scientific basis for plant drought tolerance research at the molecular mechanism level.

## Materials and methods

### Plant materials and treatments

Two sweetpotato varieties, WZ56 (drought-tolerant) and NZ2 (drought-sensitive) were obtained from Jiangsu Xuzhou Sweetpotato Research Center, P. R. China. A greenhouse pot experiment was conducted at the experimental station of Jiangsu Xuzhou Sweetpotato Research Center, Jiangsu Province, China. Healthy stem cuttings from WZ56 and NZ2 were cultured in plastic pots (20 cm deep and 17 cm diameter) filled with sandy loam soil. There were 20 pots of each variety with a single seedling in each pot). Drought treatment was conducted 10 days after transplanting. As described by Zadražnik [13], control (CK) plants were hen grown in soil that was kept at 40% soil moisture content (SMC) throughout the experiment. Seedlings in the drought stress (DS) group had irrigation withheld for ~ 20 days at which point SMC was reduced to around 8%. After treatment was completed, the third full expanded leaves from the top of CK and DS plants were used for physiological analysis and omics-experiments. Fresh leaves were flash frozen in liquid nitrogen and stored at -80°C prior to protein and metabolite extraction. For proteomics and metabolomics experiments, each biological replicate contained leaves pooled from three and six different plants, respectively.

### Measurement of relative water content, biomass and drought resistance coefficient

Freshly sampled leaves were weighed to determine the fresh weight (FW), then steeped in distilled water at 4°C for 24 h to measure the turgid weight (TW). Leaves were then oven-dried at a constant 75°C for 24 h to determine the dry weight (DW). Using these values, relative water content (RWC) was calculated as follows:

$$RWC\;\left(\%\right)=\frac{\text{FW}-\text{DW}}{\text{TW}-\text{DW}}\times 100$$ (1)

To determine biomass, CK and DS plants of both varieties were collected. Leaves were dried at 80°C until they reached a constant weight. The quantity of dry matter accumulated in the shoots was expressed in g per plant.

The drought resistance coefficient (DC) was used to quantify drought resistance among the two sweetpotato varieties as described by Hu [32].

### Determination of gas exchange parameters

The Li-6400XT portable photosynthesis system (LI-COR Biosciences, Lincoln, NE, USA) was used to detect gas exchange parameters, including net photosynthetic rate (Pn), transpiration rate (Tr), intercellular CO_2_ concentration (Ci), stomatal conductance (Gs), and instantaneous water-use efficiency (WUEi), Where WUEi = Pn/Tr.

### Measurement of photosynthesis indices

Chlorophyll was extracted from leaves and the concentrations of total chlorophyll, chlorophyll a, and chlorophyll b were calculated as previously described [33].

### Determination of proline and soluble sugar content

Proline content was determined using the quantity of colored reaction product of proline with ninhydrin as described by Bates [34]. The concentration of proline was calculated from a standard curve and expressed in μg/g of fresh weight. The colorimetric method [35] was used to determine the soluble sugar content.

### Assays of antioxidant enzyme

Test kits (Nanjing Jiancheng Biological Engineering Institute, China) were used to detect antioxidant enzyme activities, including ascorbate peroxidase (APX), superoxide dismutase (SOD), catalase (CAT) and peroxidase (POD).

### Proteomics analysis based on iTRAQ

iTRAQ analysis was performed by Shanghai Luming Biotechnology Co., LTD. This included protein preparation, iTRAQ labeling, liquid chromatography (LC)–electrospray ionization (ESI)–tandem mass spectrometry (MS/MS) analysis, protein identification and data analysis, and bioinformatics analysis.

Peptides were labeled using iTRAQ 8-plex kits (AB SCIEX, USA) according to the manufacturer^’^s protocol. The control samples were labeled with the tags 113 and 114 (NZ2) and 115 and 116 (WZ56), and the drought-stressed samples were labeled with tags 117and 118 (NZ2) and 119 and 121 (WZ56). The iTRAQ-labeled peptide mixtures were fractionated using strong cation-exchange (SCX) chromatography in the Agilent 1200 HPLC System (Agilent, Santa Clara, CA, USA) equipped with an Agilent SCX column (2.1 × 150mm, 5 μm). LC-MS/MS analysis of peptides was conducted as previously described with some modification [36]. After LC-MS/MS analysis, the MS/MS data were processed with Protein Pilot Software v.5.0 (AB SCIEX, USA) against the *Solanales* database using the Paragon algorithm [37]. Protein identification was performed with the search option. The following search parameters were used: trypsin enzyme, Carbamidomethyl (C), iTRAQ 8 plex (N-term), and deamidated (NQ) as fixed modifications, Gln-pyro-Glu (N-term Q), oxidation (M), and deamidated (NQ) as variable modifications. One max missed cleavage was allowed. The intact peptide mass tolerance was ±0.05 Da, and the fragmented ions mass tolerance was 0.1 Da. Unused >1.3 and peptides ≥2 were used as the credible protein screening criteria. Based on the average value of repeat relative quantitative values from two comparison group samples, the differentially expressed proteins (DEPs) were defined as those with fold-change > 2.0 or < 0.5.

Functional annotation of DEPs was performed using Gene Ontology (http://www.geneontology.org). The COG (http://www.ncbi.nlm.nih.gov/COG/) database was applied to functional classification of DEPs, and the Kyoto Encyclopedia of Genes and Genomes (KEGG) (http://www.kegg.jp/) was used to predict enriched pathways among DEPs. Pathways with a *p*-value < 0.05 were considered significantly enriched Cytoscape (http://www.cytoscape.org) and the string database (http://string-db.org) were used to assess and integrate of protein-protein interactions.

### Metabolomics analysis based on GC-MS and LC-MS

LC-MS and gas chromatography -MS (GC-MS) were used to analyze metabolites. Metabolites were extracted from ~80 mg of frozen leaf tissue per sample, with six biological replicates. GC-MS and LC-MS were conducted as previously described [38–40]. The differentially expressed metabolites (DEMs) between samples were screened using a combination of Orthogonal Partial least squares Discriminant Analysis ((O)PLS-DA) and Student’s *t*-test for GS-MS (VIP >1, *p* < 0.05) and LC-MS (VIP > 2, *p* < 0.01, fold change>2). Metabolites were annotated using the HMDB databases (http://www.hmdb.ca/), METLIN databases (https://www.sciencedirect.com/topics/biochemistry-genetics-and-molecular-biology/metlin), the NIST11 standard spectral databases (https://webbook.nist.gov/chemistry/quant-ir/), and Fiehn databases linked to Chroma TOF software.

### Statistical analysis

Data were analyzed using SPSS v20.0 (SPSS Inc, Chicago, IL, USA). Statistically significant differences were determined with Student’s t-test using the threshold values of *p* < 0.05 and *p* < 0.01.

## Results

### Morphological, physiological and biochemical responses under drought stress

**Fig 1:**
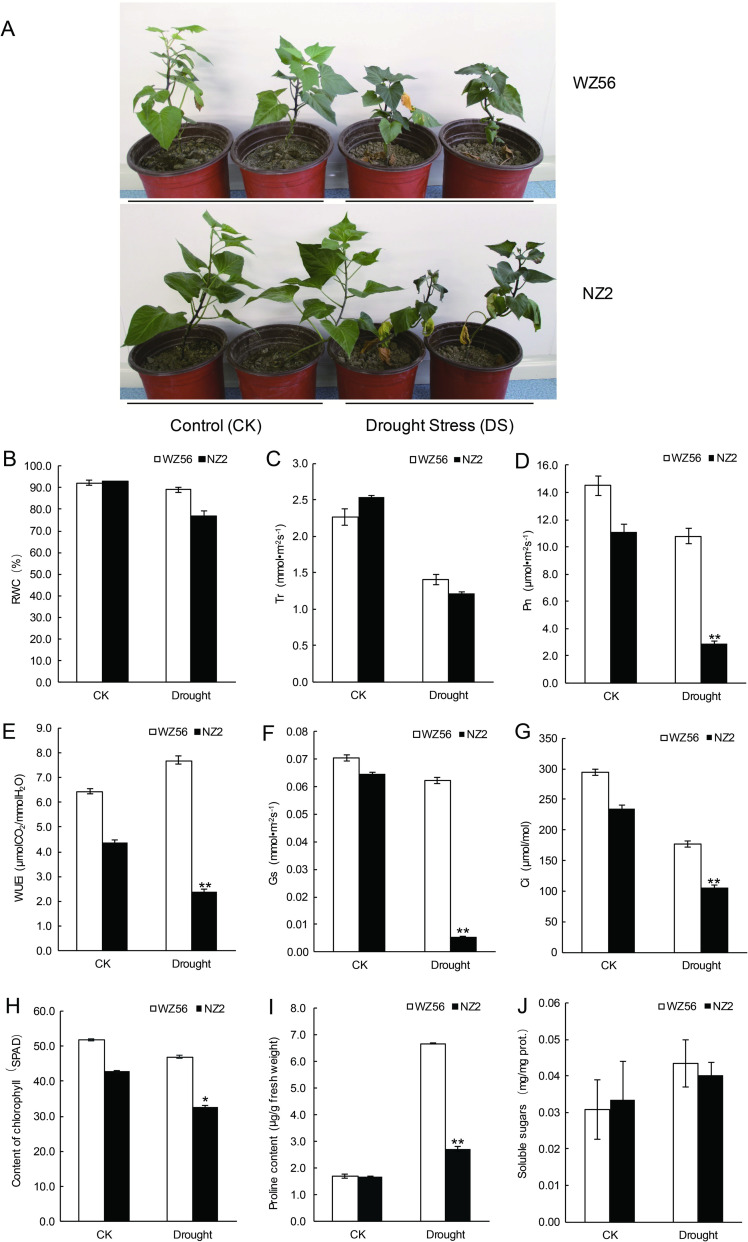
Morphological, physiological, and biochemical changes in WZ56 and NZ2 under drought stress. (A) WZ56 showed enhanced tolerance to drought compared to NZ2, (B-G) Comparison of parameters between control and drought-stressed plants and between WZ56 and NZ2 varieties. Parameters measured were (B) relative water content (RWC), (C) transpiration rate (Tr), (D) net photosynthesis rate (Pn), (E) instantaneous water-use efficiency (WUEi), (F) stomatal conductance (Gs), (G) intercellular CO2 concentration (Ci), (H) total chlorophyll concentration, (I) proline content, and (J) soluble sugar concentration. The asterisks indicate statistical significance between the WZ56 and NZ2 varieties. *p < 0.05, ** *p* < 0.01 (Student’s *t*-test).

To investigate plant responses to drought stress, morphological and gas exchange parameters and RWC were determined under control and drought conditions. The DC values of the drought-tolerant and drought-sensitive varieties (WZ56 and NZ2) were 0.93 and 0.46, respectively (S1 Fig). WZ56 showed much higher drought tolerance and lower stress responses than NZ2. Under control conditions, there was no significant difference in growth between WZ56 and NZ2, all grew rapidly and had bright green leaves. The RWC of WZ56 and NZ2 leaves was 92.3% and 92.8% respectively (Fig 1A and 1B). Growth of both WZ56 and NZ2 was inhibited and leaves were wilting after 20 days of drought stress (Fig 1A). Although, RWC in the leaves was decreased in both WZ56 and NZ2 after drought stress, it was ~13% higher in WZ56 than in NZ2 (Fig 1B). In addition, the Pn, Tr, WUEi, Gs, and Ci values of WZ56 under drought stress were 10.79 μmol/(m^2^·s), 1.41 mmol/(m2·s), 7.67 μmol CO_2_/mmol H_2_O, 0.06 mmol/(m2·s), and 177.21 μmol/mol^2^, respectively. Compared to WZ56, these values were decreased in NZ2 by 50–70% under drought stress (Fig 1C–1G).

Compared to control conditions, the chlorophyll content of WZ56 and NZ2 decreased by 10.1% and 24.5%, respectively, under drought stress (Fig 1H). Under control conditions, the proline content was 1.68–1.70μg/g in both genotypes (Fig 1I). After 20 days of drought stress, the proline content of WZ56 and NZ2 was increased by 272.2% and 77.3%, respectively (Fig 1I), representing a significant difference between the two genotypes. The variation in soluble sugar content under drought stress was similar to that of proline (Fig 1J).

### Metabolic profiles of two sweetpotato varieties under different moisture conditions

**Fig 2:**
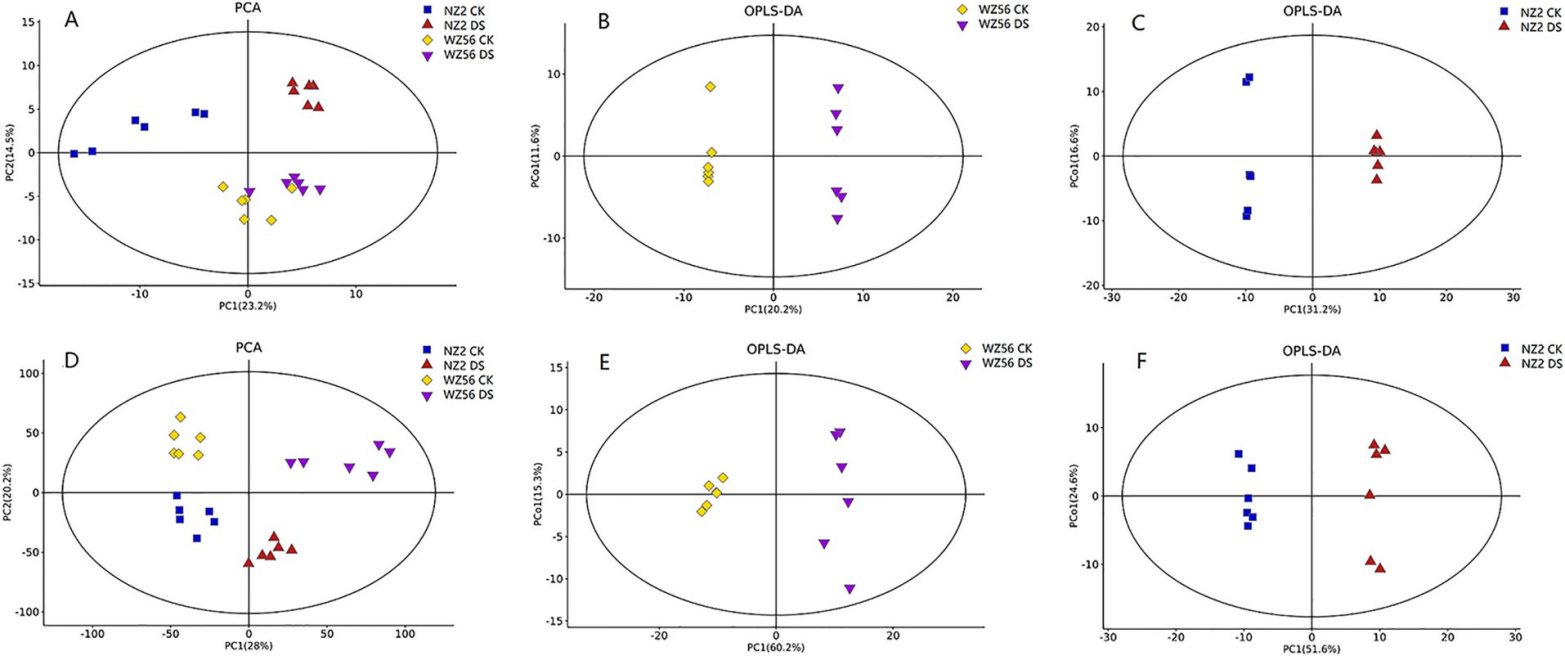
Principal component analysis (PCA) and partial least squares discriminant analysis (OPLS-DA) of WZ56 and NZ2 leaf metabolic profiles under control (CK) and drought stress (DS) conditions. (A) PCA plot for all samples (GC-MS), (B) OPLS-DA score plot between CK and DS for WZ56 (GC-MS), (C) OPLS-DA score plot between CK and DS for NZ2 (GC-MS), (D) PCA plot for all samples (LC-MS), (E) OPLS-DA score plot between CK and DS for WZ56 (LC-MS), (F) OPLS-DA score plot between CK and DS for NZ2 (LC-MS).

In total, more than 4000 metabolites were identified in NZ2 and WZ56 sweetpotato leaves under CK and DS. Principal component analysis (PCA) showed a significant shift in metabolites profile clustering in NZ2 and WZ56 groups under different moisture conditions (Fig 2A and 2D), indicating significant changes in response to drought stress.

**Fig 3:**
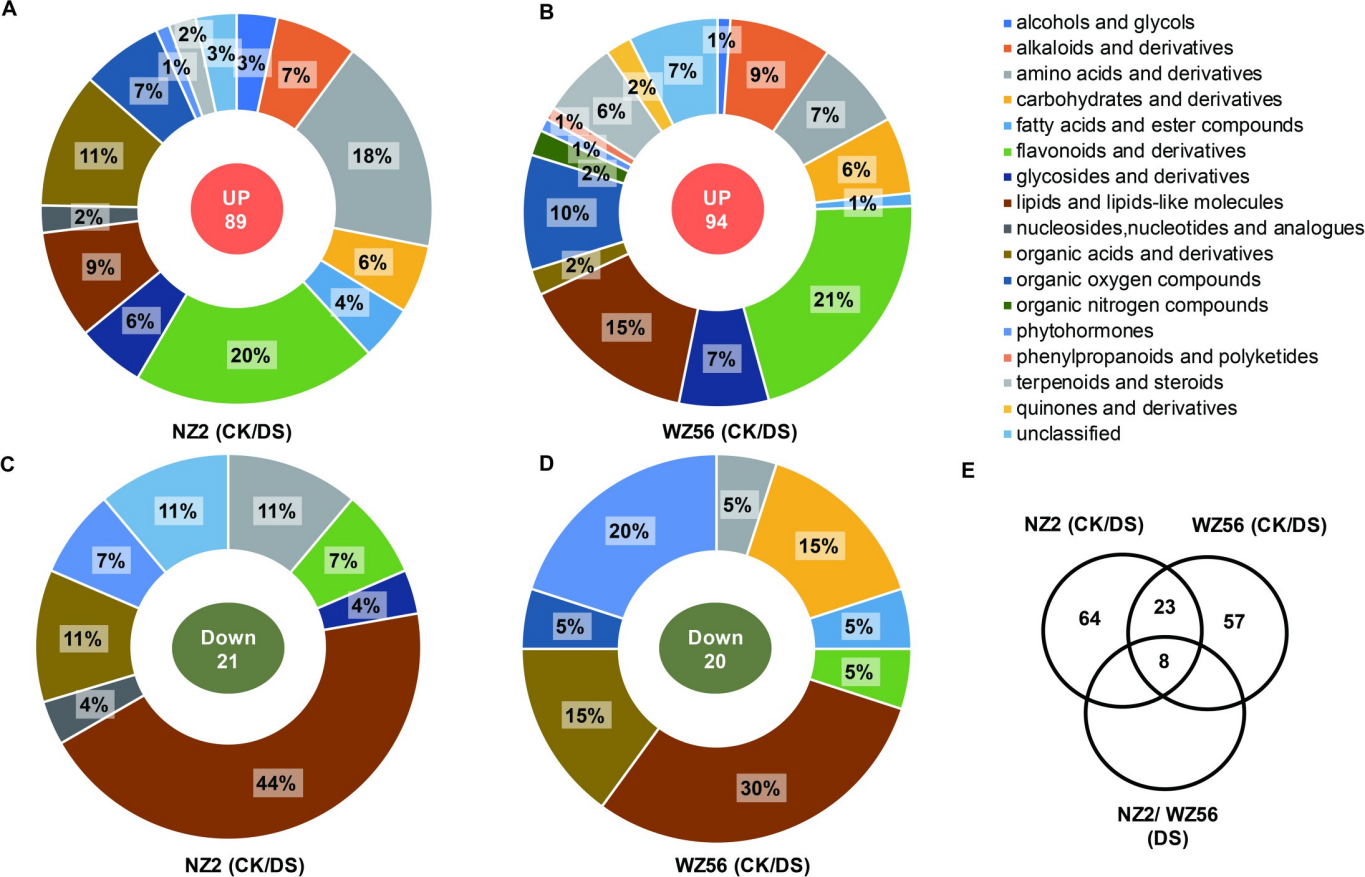
Differentially expressed metabolites (DEMs) between two sweetpotato varieties under control (CK) and drought stress (DS) conditions. (A) Up-regulated DEMs in NZ2-DS vs. NZ2-CK, (B) Up-regulated DEMs in WZ56-DS vs. WZ56-CK, (C) Down-regulated DEMS in NZ2-DS vs. NZ2-CK, (D) Down-regulated DEMS in WZ56-DS vs. WZ56-CK, (E) Venn diagram of DEMs in WZ56 CK vs. DS, NZ2 CK vs. DS, and WZ56 vs. NZ2 DS. Detailed information about the DEMs is shown in S1 and S2 Tables.

The OPLS-DA statistical analysis models (Figs 2B, 2C, 2E and 2F and 3) identified 114 and 116 differentially expressed metabolites (DEMs) in WZ56 and NZ2, respectively, between the CK and DS conditions. For WZ56, 94 of the DEMs were up-regulated in CK compared to DS and 20 were down-regulated (S1 Table), for NZ2, 89 were up-regulated and, 27 down-regulated (S2 Table). Derivatives of flavonoids and organic acids, lipid metabolites, and amino acids accounted for the highest percentage of DEMs (Fig 3A–3D). Among them, 23 DEMs co-expressed in WZ56 and NZ2 under drought stress (11.6%) could be used as potential biomarkers of drought stress (Fig 3E). The co-expressed DEMs were enriched in several metabolic pathways, including biosynthesis of plant hormones, phenylalanine-tyrosine and tryptophan biosynthesis, alpha-Linolenic acid metabolism, and aminoacyl-tRNA biosynthesis (S3 Table).

### Differential metabolic response of two sweetpotato varieties under drought stress

**Fig 4:**
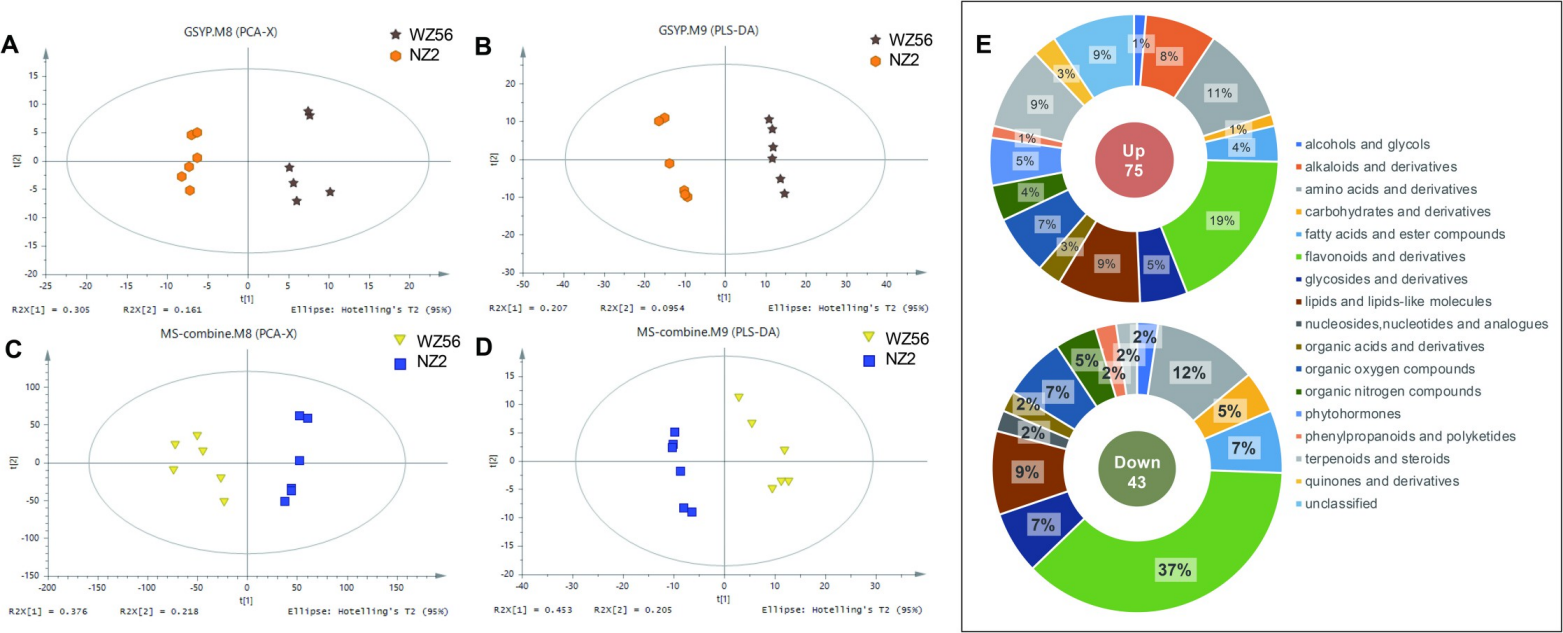
PCA and OPLS-DA analysis of metabolite profiles and DEMs functional annotations. (A) PCA for WZ56 and NZ2 metabolite samples analyzed with GC-MS, (B) OPLS-DA for WZ56 and NZ2 metabolite samples analyzed with GC-MS, (C) PCA for WZ56 and NZ2 metabolite samples analyzed with LC-MS, (D) OPLS-DA for WZ56 and NZ2 metabolite samples analyzed with LC-MS, (E) DEMs between WZ56-DS and NZ2-DS.

PCA of WZ56 and NZ2 samples under drought stress showed a significant shift in the clustering of metabolites profiles, indicating significant metabolic differences in response drought stress condition (Fig 4A and 4C). The OPLS-DA analysis models based on LC-MS showed a total of 91 DEMs (*p* < 0.05) in the leaves of WZ56 compared to NZ2 under drought stress (Fig 4B). Among them, 61 were increased and 30 were decreased (S4 Table). In addition, 27 DEMs were identified by GC-MS, with 14 increased and 13 decreased (S4 Table).

Most notably, flavonoids and derivatives, lipids and lipids-like molecules, amino acid derivatives, organic oxygen compounds, terpenoids, and steroids accounted for the highest percentage of these DEMs (Fig 4E). These metabolites primarily contain amino acids, organic acids, sugar, sugar alcohol, and fatty acid functional groups. The DEMs were enriched in pathways, including biosynthesis of plant hormones, alpha-Linolenic acid metabolism, biosynthesis of alkaloids derived from histidine and purine, biosynthesis of unsaturated fatty acids, C5-branched dibasic acid metabolism, fatty acid biosynthesis and cysteine and methionine metabolism (S3 Table). The results suggest that these metabolic pathways play an important role in sweetpotato response to drought stress.

### Identification and bioinformatic analysis of differentially expressed proteins

To further compare differences between WZ56 and NZ2 responses to drought stress, a proteomic study based on iTRAQ labeling and 2D LC-MS/MS analysis was applied to detect protein abundance changes in leaves. A total of 1140 proteins (unused >1.3 and unique peptide ≥2) were identified in these samples (S5 Table).

DEPs were defined as those with a foldchange > 2 or < 0.5. Based on this criteria and comparative proteomic analysis, 192 DEPs were found under drought treatment in WZ56 compared to NZ2 (Tables 1 and S6). Among these DEPs, 97 proteins were up-regulated, and 95 were down-regulated.

**Table 1:** Selected differentially expressed proteins (foldchange >4) between WZ56 and NZ2 under drought stress.

Accession^a^	Sequence coverage (%)	Peptides (95%)^b^	Protein Name^c^	Fold^d^
gi|404351727	78.1	92	sporamin [Ipomoea batatas]	22.79
gi|6002682	48.2	20	anionic peroxidase swpa2 [Ipomoea batatas]	16.42
gi|970009621	14.6	3	stem-specific protein TSJT1 [Solanum pennellii]	10.51
gi|37783279	52.5	11	anionic peroxidase swpb3 [Ipomoea batatas]	7.19
gi|282935442	21.4	6	anionic peroxidase [Ipomoea batatas]	6.40
gi|697132539	29.8	5	L-ascorbate peroxidase 2, cytosolic isoform X2 [Nicotiana tomentosiformis]	5.57
gi|33516947	51.7	42	RecName: Full = Anionic peroxidase; AltName: Full = SwPA1; Flags: Precursor	5.54
gi|565379315	61.8	77	chlorophyll a-b binding protein 3C, chloroplastic-like [Solanum tuberosum]	5.38
gi|697121824	17.1	3	peroxidase P7-like [Nicotiana tomentosiformis]	5.35
gi|302584050	71.5	211	chloroplast LHCII type I chlorophyll a-b binding precursor protein, partial [Ipomoea nil]	5.31
gi|85690845	11.3	3	aquaporin-like protein [Ipomoea nil]	5.02
gi|697127312	48.7	28	chlorophyll a-b binding protein 8, chloroplastic-like [Nicotiana tomentosiformis]	4.71
gi|75263813	19.9	6	RecName: Full = Peroxidase 15; Short = Prx15; AltName: Full = Anionic peroxidase; Flags: Precursor	4.70
gi|697112735	4.1	2	leucine—tRNA ligase, cytoplasmic-like [Nicotiana tomentosiformis]	4.70
gi|966873208	36.7	305	putative photosystem II CP43 chlorophyll apoprotein-like [Solanum chacoense]	4.66
gi|966859522	9.3	4	putative asp precursor-like [Solanum chacoense]	4.54
gi|66876475	45.3	184	PsbC (chloroplast) [Cuscuta sandwichiana]	4.42
gi|697177674	19	11	alpha-xylosidase 1-like [Nicotiana tomentosiformis]	4.16
gi|33516948	35.1	26	RecName: Full = Neutral peroxidase; AltName: Full = SwPN1; Flags: Precursor	3.89

^a^ Protein gi number from NCBI.

^b^ The total number of detected peptides (with 95% confidence) for the individual protein species.

^c^ Name of the protein identified by MS/MS.

^d^ The protein expression ratio in WZ56 to NZ2 leaves under drought stress.

**Fig 5:**
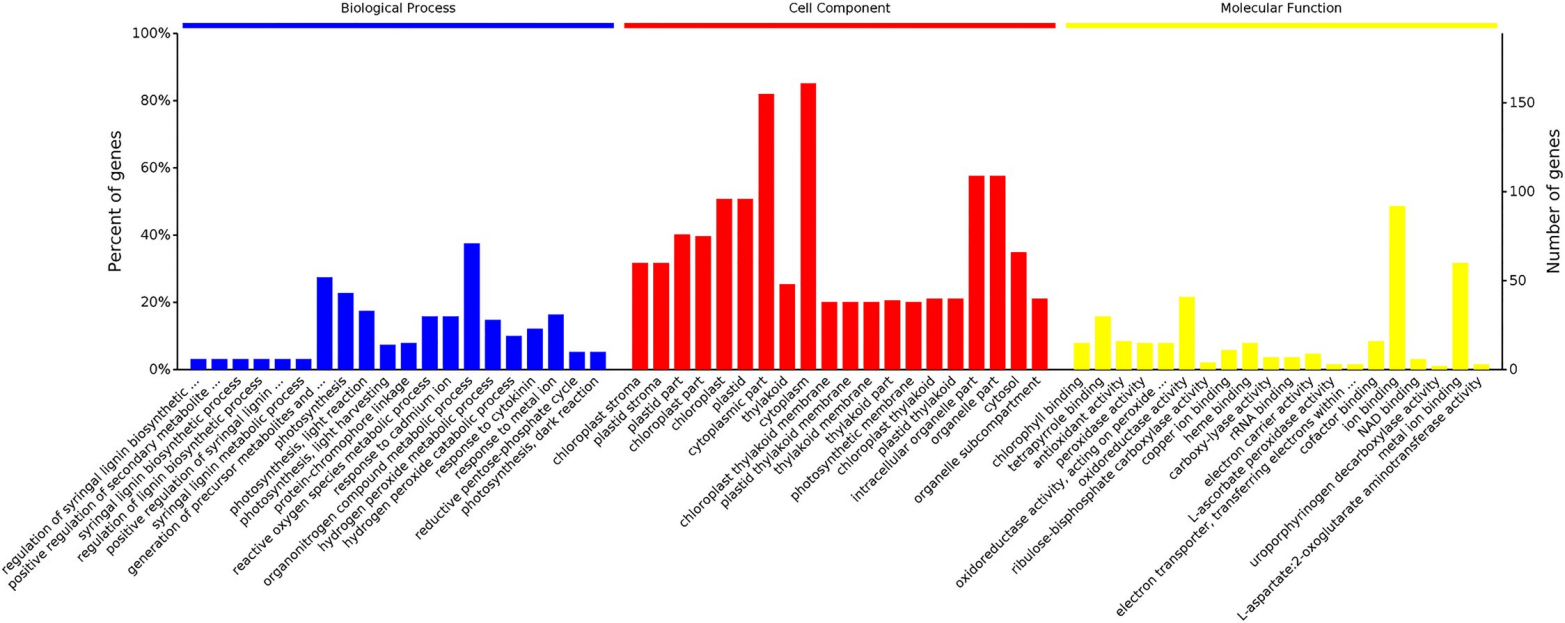
The 20 most enriched biological process, cell component, and molecular function annotations in the 192 DEPs.

**Fig 6:**
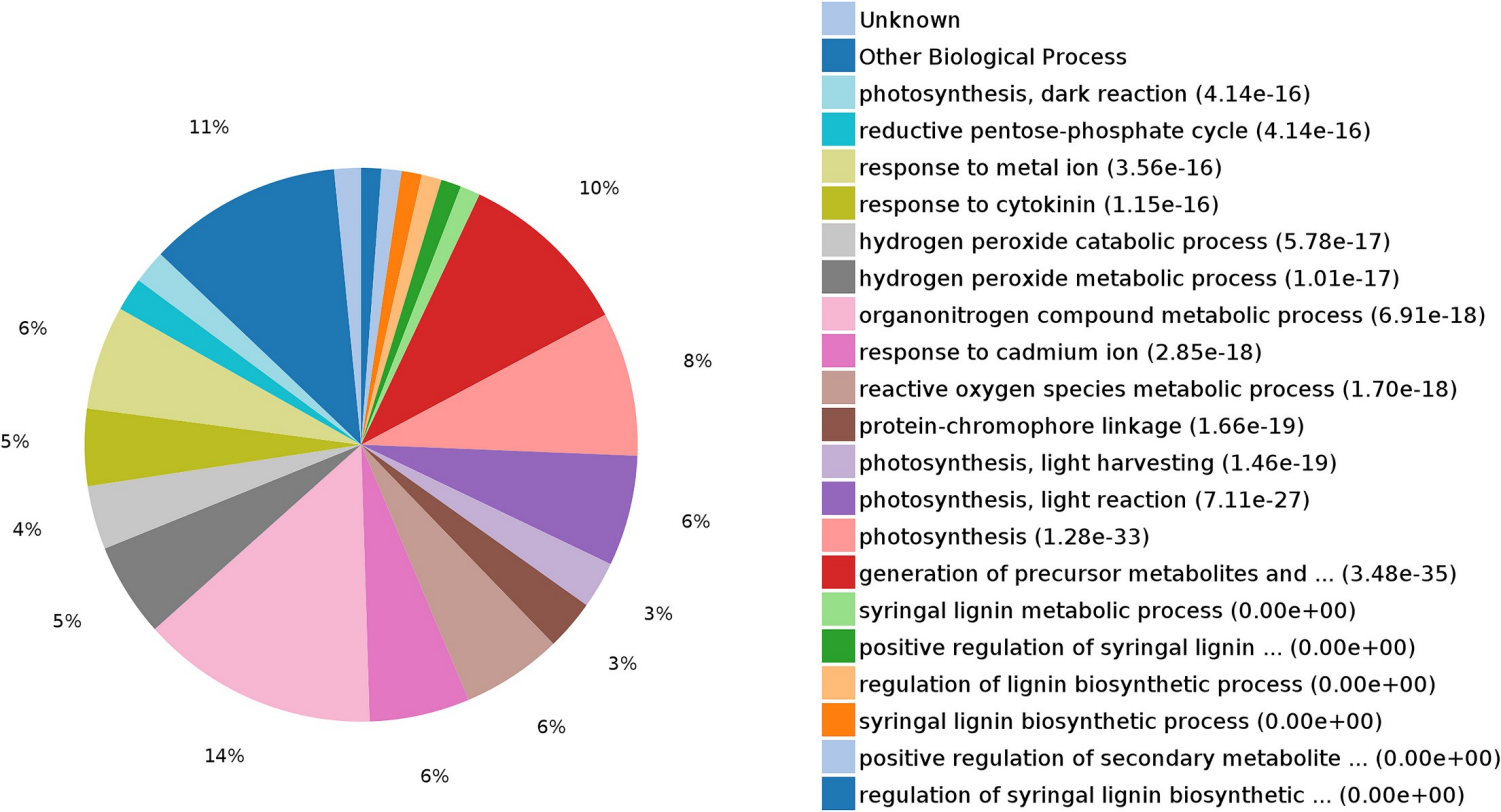
Functional classification of the 192 DEPs in WZ56 vs. NZ2 under drought stress.

Proteins were annotated using GO terms to identify significantly enriched functions in the DEPs. Annotation categories comprised biological processes (BP), cell components (CC), and molecular functions (MF). The 20 highest -ranked annotations from each category at the 1% significance level are shown in Fig 5. Significantly enriched CC terms were cytoplasm, intracellular organelle, plastid, and chloroplast; for MF, enriched terms were ion binding, metal ion binding, oxidoreductase activity, and tetrapyrrole. These DEPs were enriched in a wide range of BP terms, including organonitrogen compound metabolic process (14%), generation of precursor metabolites and energy (10%), photosynthesis (8%), photosynthesis-light reaction (6%), photosynthesis-light harvesting (3%), reactive oxygen species metabolic process (6%), response to cadmium ion (6%), hydrogen peroxide metabolic process (5%), and hydrogen peroxide catabolic process (4%) (Fig 6). The analysis showed that some proteins involved in different biology process under drought stress, including ascorbate peroxidase (gi|953834935), chlorophyll a-b binding protein 21 (gi|698478410), peroxidase P7-like (gi|697121824), PsbC (chloroplast) (gi|66876475), ribulose-1,5-bisphosphate carboxylase/oxygenase large subunit (gi|608608638), cysteine protease (gi|13897890), and Mn-SOD (gi|1160982).

**Fig 7:**
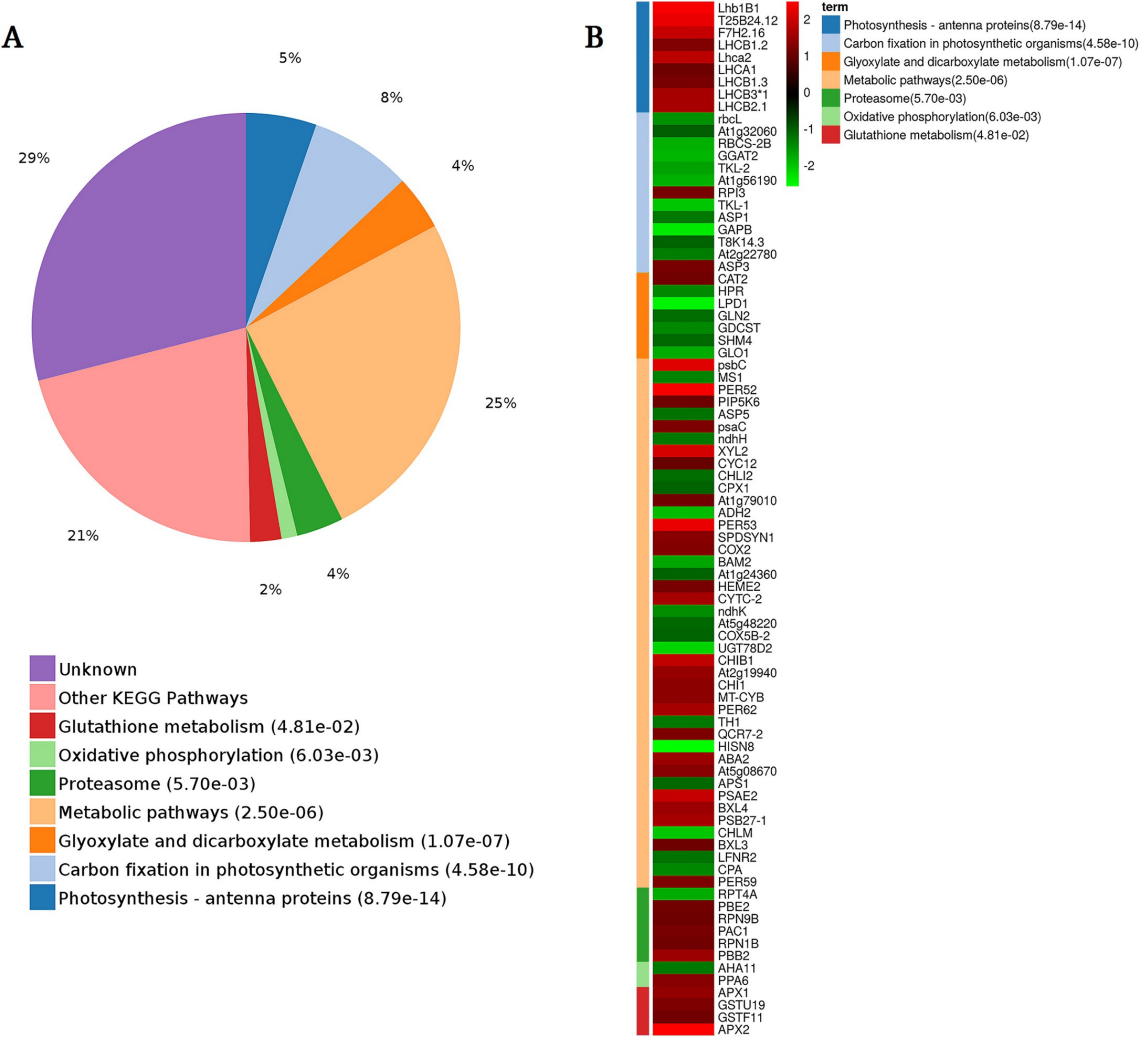
Significantly enriched KEGG pathways among DEPs. (A) KEGG pathway annotations for the 192 DEPs in WZ56 compared to NZ2 under drought stress, (B) Expression level of DEPs in the enriched pathways.

KEGG pathway analysis was also performed on the 192 DEP, and indicated that they were significantly enriched in metabolic pathways (25%), carbon fixation in photosynthetic organisms (8%), photosynthesis-antenna proteins (5%), glyoxylate and dicarboxylate metabolism (4%), proteasome (4%), glutathione metabolism (2%), and oxidative phosphorylation (1%) (Fig 7A). All DEPs related to photosynthesis and glutathione metabolism were up-regulated under drought stress, indicating a possible role of photosynthesis and glutathione metabolism in sweetpotato response to drought stress. Most of the DEPs related to the proteasome and metabolic pathways were also up-regulated. In contrast, DEPs related to the carbon fixation in photosynthetic organism pathway, glyoxylate pathway, and dicarboxylate metabolism pathway were primarily down-regulated (Fig 7B).

### Bioinformatics analysis of DEPs and DEMs based on KEGG pathways

**Fig 8:**
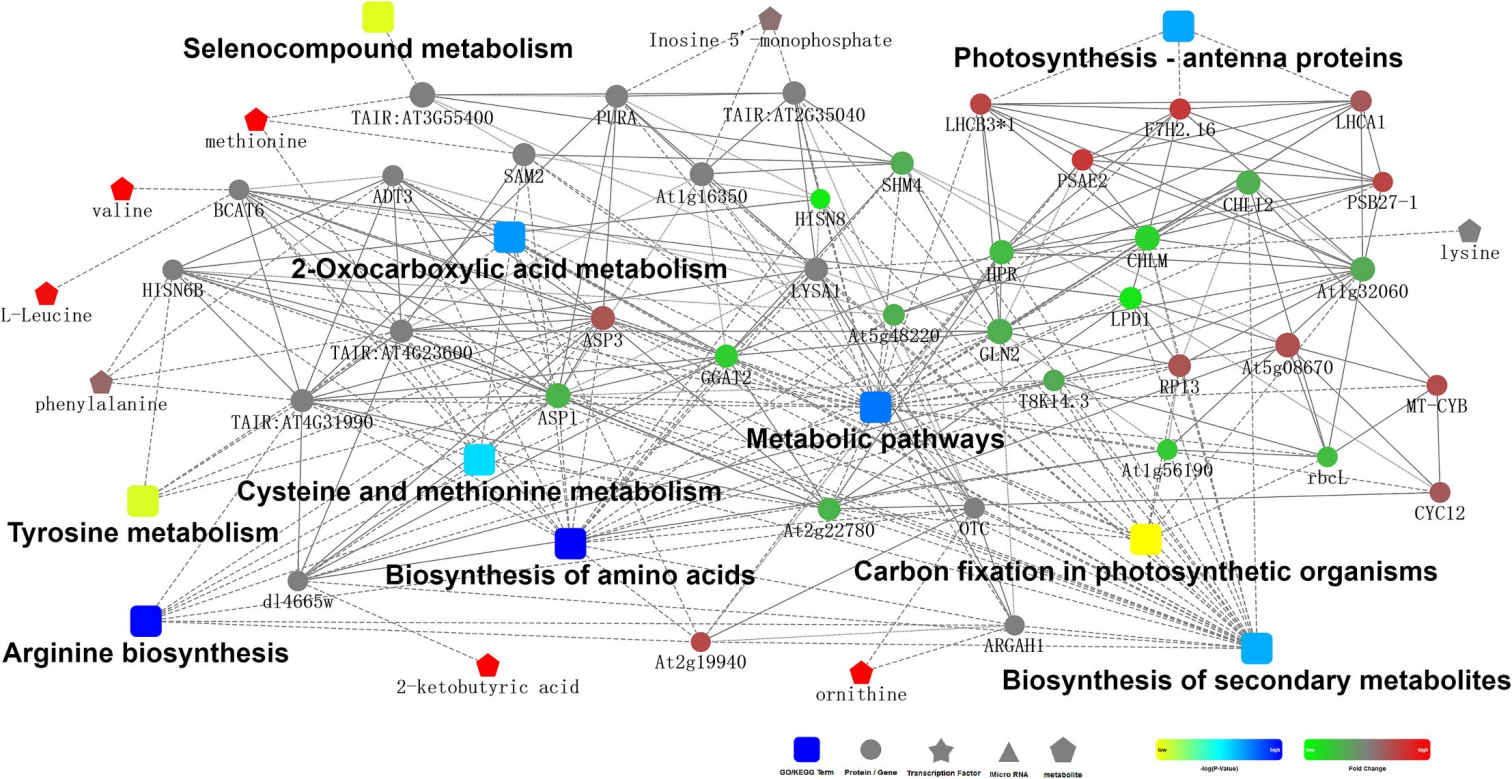
Interaction network of DEPs and DEMs with KEGG pathway annotations.

KEGG pathway analysis was conducted to integrate the DEPs and DEMs datasets for both genotypes under drought stress. There were 28 pathways enriched in the DEPs and DEMs. some of those pathways met the threshold for being considered significantly enriched (*p*- value < 0.05), including metabolic pathways, biosynthesis of amino acids, biosynthesis of secondary metabolites, phenylpropanoid biosynthesis, and 2-Oxocarboxylic acid metabolism (S3 Table). There were complicated interactions between DEPs, DEMs, and KEGG pathways (Fig 8). The DEPs and DEMs related to biosynthesis of amino acids, including valine, leucine, O-acetylserine, 2-ketobutyric acid, methionine, N-acetyl-gamma-glutamyl-phosphate reductase (gi|970004241), and ribose-5-phosphate isomerase 3 (gi|970001376), were significantly up-regulated in WZ56. DEPs and DEMs related to the phenylpropanoid biosynthesis pathway, such as POD N1-like (gi|966842423) (3.0-fold higher), POD N (gi|698524586) (2.3-fold), anionic POD (gi|75263813) (4.7-fold), POD swpa2 (gi|6002682) (16.4-fold), anionic POD (gi|33516947) (5.5-fold), anionic POD swpb3 (gi|37783279) (7.2-fold), anionic POD (gi|282935442) (6.4-fold), neutral POD (gi|33516948) (3.9-fold), POD P7-like (gi|697121824) (5.4-fold), and chlorogenic acid (C00852), were also up-regulated in WZ56 compared to NZ2. This indicated that phenylpropanoid biosynthesis may play an important role in the sweetpotato response to drought stress. Additionally, higher levels of energy metabolism and defense-related proteins and metabolites in drought-stressed leaves suggested activation of reactive oxygen species (ROS) scavenging system and improvement of photosynthesis.

### Activity of antioxidant enzymes involved in the ROS scavenging system

**Fig 9:**
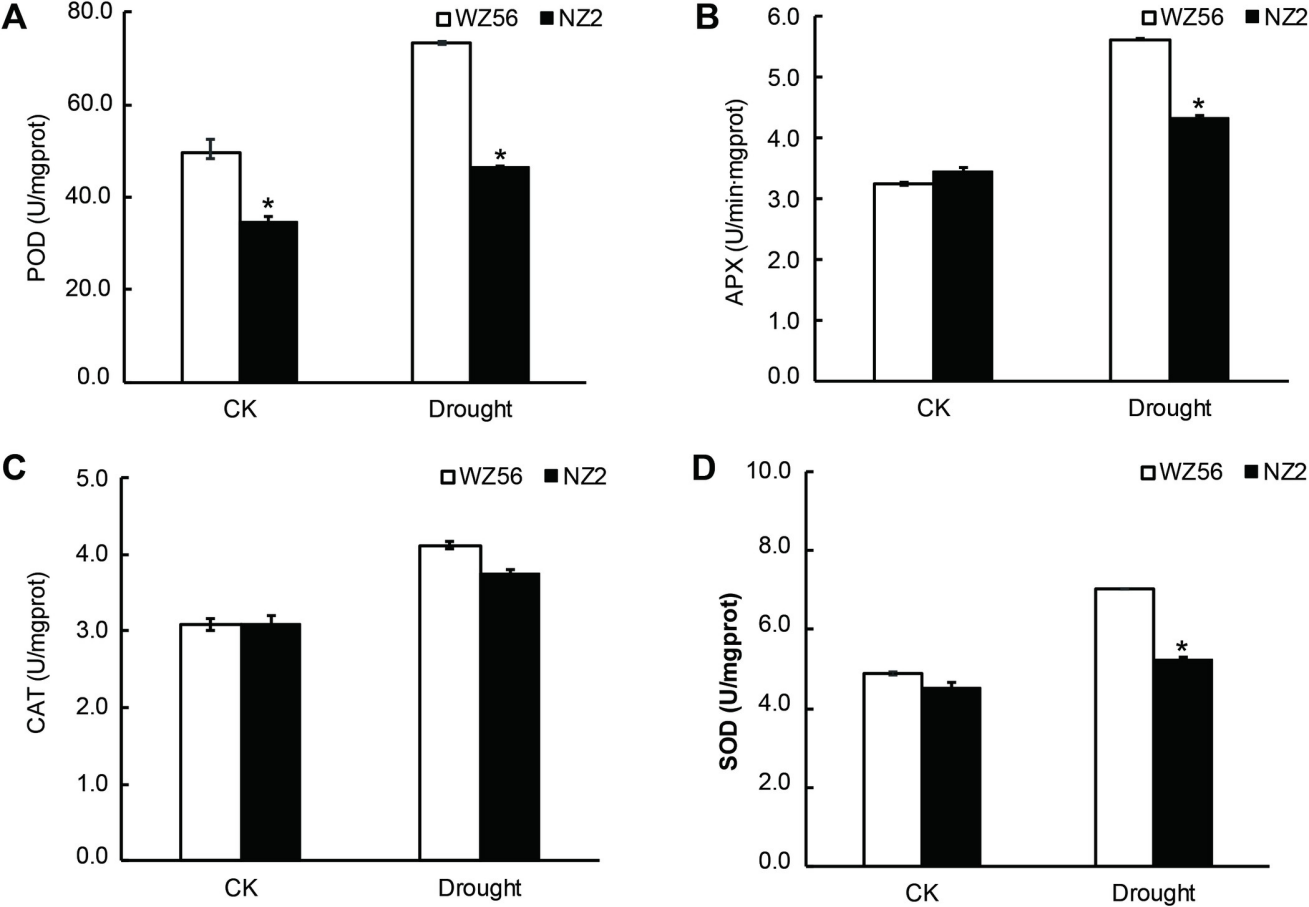
Antioxidant enzyme activities. (A) POD, (B) APX, (C) CAT, (D) SOD. CK: control conditions, with soil water content kept at ~40%; DS: drought stress conditions, with soil moisture content reduced to ~ 8% over 20 days. *p < 0.05 (Student’s t-test) WZ56 vs. NZ2 variety.

To determine the effect of drought stress on the antioxidant levels, the activities of APX, SOD, CAT and POD were quantified. No significant differences were detected in APX, CAT and SOD activity between WZ56 and NZ2 under control conditions. However, POD, APX, CAT and SOD activity increased significantly in both cultivars under drought stress, compared to NZ2, the POD, APX, CAT, and SOD activity of WZ56 increased by 58.2%, 29.2%, 9.5%, and 33.9%, respectively (Fig 9).

Proteins up-regulated in WZ56 compared to NZ2 under drought stress included four anionic PODs (gi|6002682, gi|37783279, gi|282935442, and gi|33516947), POD P7-like (gi|697121824), POD 15 (gi|75263813), neutral POD (gi|33516948), and POD N (gi|698524586). Notably, three of the anionic POD proteins were increased by a large margin ((> 6-fold). In addition, levels of an APX (gi|953834935), an L-APX 2 protein (gi|697132539), a CAT (gi|282935438), and an Mn-SOD protein (gi|1160982) were also up-regulated in WZ56 under drought stress (S6 Table). The changes in enzyme activities were consistent with the protein expression levels. This consistency demonstrates the high quality of the proteomics data.

## Discussion

Water deficit is a major limiting factor for plant growth and yield in arid areas. Enhancing plant drought tolerance will aid in overcoming this limitation, thus contributing to crop production and global food security. To decipher the molecular mechanisms underlying the response of sweetpotato to drought stress, an integrated physiological, proteomics and metabolomics study was conducted. The results showed that drought stress led to drastic changes in both the proteome and metabolome in the leaves of two sweetpotato varieties. Biological processes such as photosynthesis and regulation and metabolism of sugars, amino acids and flavonoids were found to be involved in drought tolerance in sweetpotato.

### Effects of drought stress on sweetpotato physiology

To obtain insight into the physiological response of WZ56 and NZ2 to drought stress, several physiological indicators were measured. The RWC of leaves reflects the water-reserving capacity of its cell, and can closely reflect the balance between water supply and transpiration. Here, the RWC was found to be higher in WZ56 than in NZ2 under drought stress (Fig 1B). Owing to increased osmotic regulation of the cell wall, the drought-resistant plant could maintain high RWC; a higher RWC in turn contributes to maintaining growth, photosynthesis and normal metabolic activity under water deficit [41]. This suggests that a higher RWC in leaves may help to reduce the damage caused by drought stress.

### Effects of drought stress on photosynthesis and chlorophyll metabolism of sweetpotato leaves

The proteomics results showed that some proteins involved in photosynthesis were affected by drought stress (S6 Table). RuBisCO, the first enzyme involved in catalyzing the carbon cycle and photorespiration, showed slight down-regulation in WZ56 under drought stress (S6 Table). Although the total chlorophyll content in WZ56 and NZ2 leaves was decreased under drought stress compared to the control (Fig 1H). WZ56 had higher total chlorophyll content, Pn, Tr, WUEi, Gs, and Ci compared to NZ2(Fig 1C–1F). Drought stress had significant effects on photosystem I (PS I) and photosystem II (PS II) [42]. The activity and concentration of photosynthetic carbon reduction cycle enzymes was reduced reduction by drought stress [43]. Photosynthesis is known to be inhibited by changes in chlorophyll content and damage to chloroplasts under drought stress [44]. Drought stress resulted in a decrease in stomatal conductance (Gs) and internal CO2 concentration (Ci). A decrease in carbon dioxide fixation has been shown to lead to a decrease in the photosynthetic rate [45, 46]. Our results in combination with previous findings suggest that the activity of RuBisCO is reduced under drought stress. The drought-resistant variety of sweetpotato had higher chlorophyll content, Pn, Tr, WUEi, Gs, and Ci than the drought-sensitive variety, suggesting that drought-resistant variety could maintain higher rates of photosynthesis than drought-sensitive variety in response to water deficit.

The proteomics analysis revealed that many proteins involved in photosynthesis were up-regulated in the drought-resistant variety under drought stress. As key components of the light harvesting system, all of the chlorophyll a/b binding proteins among the DEPs were up-regulated in drought-resistance variety under drought stress (Tables 1 and S6). The light harvesting chlorophyll a/b binding proteins act as a switch played an important role in photosystem I and photosystem II. Up-regulation of these proteins could enhance stomatal sensitivity to abscisic acid (ABA), and results in an increase in plant tolerance to drought stress [47]. The ABA signaling pathway plays a vital role in plant drought resistance; it can trigger stomatal closure to decrease water loss, and induce a series of changes in gene expression to prevent cells from oxidative damage [48]. Therefore, up-regulation of chlorophyll a/b binding proteins may contribute to capturing light energy, improving photosynthesis and drought resistance in WZ56.

We additionally found that some proteins involved in photosynthesis, (such as photosystem I reaction center subunit IV A, photosystem II 44 kDa protein, putative photosystem II CP43 chlorophyll apoprotein-like, photosystem II repair protein PSB27-H1 were significantly up-regulated in the leaves of WZ56 (Table 1) in response to drought. These results suggest that up-regulation of these proteins may contribute to improvement of photosynthesis in WZ56 under drought stress, further improving its drought resistance through photosynthetic regulation.

### Effects of drought stress on sugar metabolism in sweetpotato leaves

The metabolomics results showed that drought stress induced an increase in gentiobiose, inositol and gluconic lactone content in WZ56 and NZ2 (S1 and S2 Tables), and a significant accumulation of sucrose in WZ56 (S1 Table). Soluble sugar content showed a similar increasing trend under drought stress, and WZ56 had higher levels of soluble sugar than NZ2 (Fig 1J). Sugars are highly sensitive to environmental stresses and ensure the supply of carbohydrates from source to sink organs during stress responses. Many studies have shown that levels of glucose, fructose, and the raffinose family oligosaccharides (RFOs) increased in plants under dehydration compared with control conditions [49, 50]. Sucrose is the primary product of photosynthesis and is widely considered an energy source for metabolic activity in plants [51]. Soluble carbohydrates are compatible solutes, and their accumulation can decrease the osmotic potential in plants to ensure sufficient water for normal plant growth under drought conditions [52, 53]. They may serve as osmolytes and osmoprotectants to prevent membrane fusion and to stabilize enzymes and other cellular components in response to drought stress [54, 55]. Moreover, some metabolites, most notably sucrose and fructose, also help to protect cells from oxidative damage via osmoregulation or ROS scavenging under drought conditions [56, 57]. These metabolites accumulated significantly in the two sweetpotato varieties studied here under drought stress, implying that their accumulation was a universal and critical defense mechanism against drought in sweetpotato.

### Effects of drought stress on amino acid metabolism of sweetpotato leaves

Under control conditions, the proline content of WZ56 and NZ2 was found to be comparable. However, under drought stress, the proline content of WZ56 was significantly increased compared to NZ2 (Fig 1I). The levels of leucine, ornithine, and lysine and its precursor alpha-aminoadipic acid also increased significantly in both WZ56 and NZ2 under drought stress conditions (S1 and S2 Tables). In higher plants, amino acids accumulate in response to various stresses and have multiple functions in plant growth [58]. They are an important nitrogen source of nitrogen, and specific amino acids may be able to delay protein degradation under drought conditions [59, 60]. For example, in response to drought stress, proline protects plant cell membranes and proteins and functions as a ROS scavenger, thus enhancing plant resistance [61, 62]. Levels of the branched-chain amino acids (BCAAs) valine, leucine and isoleucine and other related amino acids such as lysine, threonine,β-alanine and methionine generally increase under stress [63]. The observed accumulation of proline and BCAAs in sweetpotato leaves under drought stress sweetpotato suggests that proline and BCAAs may be closely associated with plant drought tolerance.

Aromatic amino acids (AAAs) serve as precursors for a large number of specialized metabolites [64] and are known to increase in concentration under drought conditions. Previous studies showed that increased levels of AAAs were used in producing secondary metabolites during stress responses [58]. Additionally, phenylalanine influences osmotic adjustment and is a precursor to many key secondary metabolites (e.g. phenylpropanoids, flavonoids, catechin and kaempferol) that contribute to plant drought tolerance [65, 66]. Under drought stress, the levels of L-phenylalanine, L-tryptophan and L-tyrosine were increased in WZ56 and NZ2 compared to the control conditions (S1 and S2 Tables). This significant drought-induced accumulation of phenylalanine implies that this amino acid is important for sweetpotato drought tolerance.

### Effects of drought stress on phenylpropanoid biosynthesis and metabolism of sweetpotato leaves

In this study, DEPs and DEMs related to the phenylpropanoid biosynthesis pathway were found to be significantly up-regulated in WZ56. These DEPs and DEMs included Polyphenol oxidase I, POD 15, beta-glucosidase 44-like, and chlorogenic acid (S5 and S7 Tables). Phenylpropanoids contribute substantially to the stability and robustness of plant responses to biotic and abiotic stress [64]. Drought stress could increase expression of genes involved in the phenylpropanoid biosynthesis pathway [67]. In addition, drought stress induces the accumulation of phenylpropanoids in the vacuoles of mesophyll cells, these phenylpropanoids may constitute a secondary antioxidant system to scavenge H_2_O_2_ [68]. Chlorogenic acid is a common soluble phenylpropanoid in the Solanaceae, and acts as an antioxidant [69, 70]. The response of members of the phenylpropanoid biosynthesis pathway to drought stress indicated co-regulation of DEPs and DEMs in WZ56.

Flavonoids, another type of phenylpropane compound, are a class of important secondary metabolites that are widely present in plants. Previous studies have shown that flavonoids exert antioxidant and ROS scavenging effects [71–73]. Here, we found that many flavonoids were differentially expressed under drought stress. 20 and 21 in NZ2 and WZ56, respectively, most of which were up-regulated (S1 and S2 Tables). These results suggest that moderate drought stress increased accumulation of flavonoids in sweetpotato.

### Effect of drought stress on antioxidant levels of sweetpotato leaves

Proteomic analysis showed that proteins significantly up-regulated in response to drought stress, included L-APX 2, POD P7-like, putative POD N1-like, peroxiredoxin, Mn-SOD, APX, POD N, CAT, probable glutathione S-transferase parC, and glutathione S-transferase-like (S5 Table). These results indicated that co-regulation of these antioxidant enzymes may contribute to drought stress resistance in WZ56. Drought stress can induce oxidative stress in plants. Plants can either eliminate or reduce ROS-induced injury by regulating antioxidant content, which is an effective mechanism of stress resistance [74]. Drought stress may stimulate excessive ROS production, which results in oxidative damage to cellular components [75]. SOD is generally considered the first line of defense against oxidative stress via conversion of highly toxic superoxide to less toxic hydrogen peroxide. The ascorbate-glutathione cycle is a major plant hydrogen peroxide detoxifying system, in which APX is a vital enzyme [76] We found that POD was significantly up-regulated in leaves under drought stress. The major function of POD is hydrogen peroxide hydrolysis to relieve cell damage. Glutathione S-transferases (GSTs) comprise a major family of multifunctional enzymes that plays a key role in the process of plant detoxification [77, 78]. A conjugate of glutathione (GSH) and cytotoxin is known to be transferred to the vacuole or expelled in vitro after catalysis by GST [79]. Overexpression of a GST gene from wild soybean significantly enhanced drought tolerance in transgenic tobacco [80]. The results of the present study are consistent with those of previous studies, suggesting that drought stress may induce increased antioxidant enzyme activities to scavenge ROS. Enzyme activity assays were consistent with the results from proteomics verifying the reliability of the proteomic results (Fig 9).

### Effects of drought stress on energy metabolism and other metabolism in sweetpotato leaves

Expression of the ATP synthase beta subunit was found to be significantly increased in WZ56 compared to NZ2 under drought stress (S6 Table). ATP synthase is closely related to energy metabolism; it accelerates synthesis of ATP for photosynthetic activities. ATP synthase is a vital link in electron transport between PS I and PS II [81], and plays a significant role in regulating photosynthetic electron flow in higher plants [82, 83]. It participates in removing damaged proteins, protecting key components, and has a chaperone and peptidase activity [84]. The high—expression levels of ATP synthase could provide the energy required for resistance to drought stress [85]. Therefore, the up-regulation of ATP synthase in WZ56 may contribute to increase drought stress resistance.

Compared with NZ2, acid phosphatase activity was significantly increased in WZ56 under drought stress (S5 Table). The putative harpin binding protein 1-like and aquaporin-like protein were also found to be significantly increased in WZ56 leaves under drought stress (S5 Table). In wheat, a significant increase in acid phosphatase activity was detected in leaves of a drought tolerant cultivar, whereas no change was observed in a drought-sensitive cultivar [86]. Overexpression of a harpin-encoding gene in tobacco plants increased tolerance to drought stress [87]. Exogenous application of harpin significantly activated defense pathway mediated by jasmonic acid and salicylic acid [88]. Aquaporin-like protein may improve plant drought resistance by decreasing transpiration via reducing stomatal conductance [89]. Our results suggest that the up-regulation of these DEPs may enhance resistance to drought stress by improving defense mechanisms.

In the present study, significant up-regulation of the putative cysteine protease and putative cysteine proteinase 3-like proteins in WZ56 was observed under drought stress (S6 Table). As a protease family, cysteine protease participated in various physiological processed of plant widely. Some cysteine proteases localized in chloroplast and vacuole are capable of degrading the RuBisCO large subunit, and play an important role in leaves under drought stress [90]. Cysteine proteases also play an important role in programmed cell death [91]. Constitutive expression of sweetpotato cysteine protease (SPCP2) in Arabidopsis significantly increases tolerance to salt and drought stress [92]. Transgenic Arabidopsis expressing cysteine protease from *Salix matsudana* showed a higher tolerance to salt compared to the control [93]. Cysteine proteases are mainly localized in the chloroplasts and nuclei, its expression unrequired ABA, and are related to drought-induced senescence and programmed cell death. These results indicate that cysteine proteases may play a key role in tolerance to drought stress in WZ56. In response to drought stress, cysteine protease activity may inhibit RubisCO, or interact with chlorophyll a/b binding proteins in chloroplasts. This drought-resistance mechanism requires further study.

## Conclusions

In this study, a comparative analysis was conducted using physiological measurements, proteomics and metabolomics to reveal the physiological and molecular responses of sweetpotato to drought stress. Sweetpotato cultivars WZ56 and NZ2 were used, which are drought-tolerant and drought-sensitive, respectively. Based on physiological measurements, the drought-tolerant variety (WZ56) had better water retention capacity and water use efficiency under drought stress condition. Proteomic analysis showed that sweetpotato responded to drought stress by changing the expression pattern of a number of proteins involved in photosynthesis, ROS metabolism, organonitrogen compound metabolism, and generation of precursor metabolites and energy. In particular, 192 proteins were identified as differentially expressed, 97 of which were up-regulated by drought stress in WZ56 but not in NZ2. This difference indicated that these DEPs may play an important role in tolerance to drought stress in WZ56. Metabolomics analysis revealed that 75 metabolites were increased in WZ56 but decreased in NZ2, many of which were involved in alkaloid metabolism or biosynthesis of flavonoids and derivatives, amino acids, unsaturated fatty acids, fatty acid s, or plant hormones (S5 Table). Up-regulated DEPs and DEMs in WZ56 were significantly enriched in the phenylpropanoid biosynthesis pathway. This study presents specific proteins and metabolites associated with drought tolerance, and furthermore provides insights into the physiological, proteomic and metabolomic basis for drought tolerance in sweetpotato.

## Supporting information

S1 Fig The drought resistance coefficient of WZ56 and NZ2. (TIF)

S1 Table DEMs of WZ56, CK vs. DS. (XLSX)

S2 Table DEMs of NZ2, CK vs. DS. (XLSX)

S3 Table Enrich metabolic pathways of three different grouping. (XLSX)

S4 Table DEMs of WZ56 DS vs. NZ2 DS. (XLSX)

S5 Table 1140 proteins with different protein abundances. (XLS)

S6 Table Selected differentially expressed proteins in WZ56 DS vs. NZ2 DS. (XLSX)

S7 Table Pathways of common enrichment of DEPs and DEMs. (XLSX)
