# Supplementary figures and images for: Differential response of physiology and metabolic response to drought stress in different sweetpotato cultivars
Source: PLoS One. 2022 Mar 10;17(3):e0264847. doi: 10.1371/journal.pone.0264847 (PMC8912141; doi:10.1371/journal.pone.0264847)

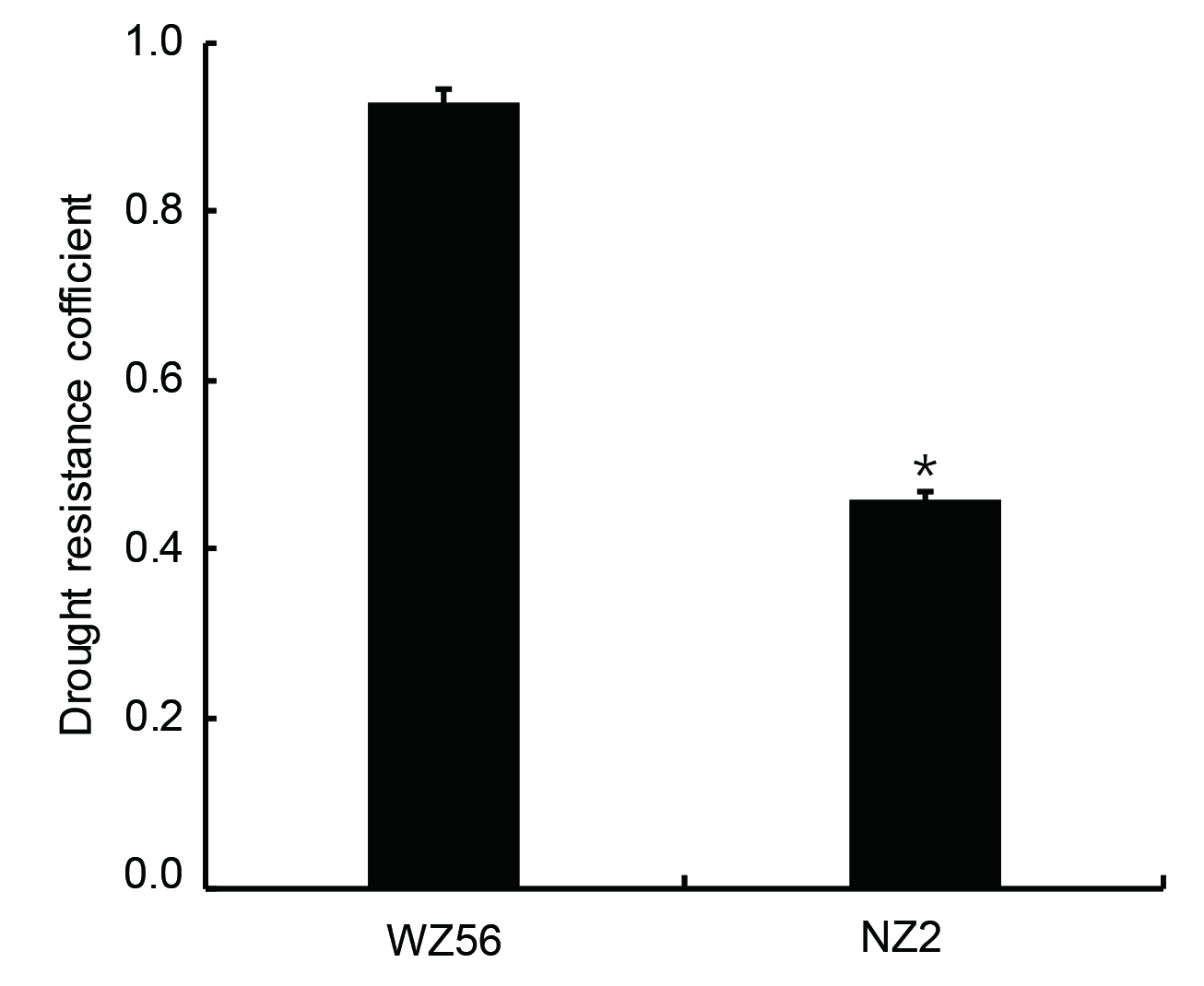

Supplement: S1 Fig — (TIF) [file pone.0264847.s001.tif]
